# Predictors of mortality in severe pneumonia patients: a systematic review and meta-analysis

**DOI:** 10.1186/s13643-024-02621-1

**Published:** 2024-08-05

**Authors:** Kai Xie, Shengnan Guan, Xinxin Kong, Wenshuai Ji, Chen Du, Mingyan Jia, Haifeng Wang

**Affiliations:** 1 Department of Respiratory Medicine, The First Affiliated Hospital of Henan University of Chinese Medicine, Zhengzhou, China; 2 Academy of Chinese Medical Sciences, The First Clinical Medical College of Henan University of Chinese Medicine, Zhengzhou, China; 3 Co-Construction Collaborative Innovation Center for Chinese Medicine and Respiratory Diseases By Henan & Education Ministry of P.R. China, Henan University of Chinese Medicine, Zhengzhou, China

**Keywords:** Severe pneumonia, Systematic review, Mortality

## Abstract

**Background:**

Severe pneumonia has consistently been associated with high mortality. We sought to identify risk factors for the mortality of severe pneumonia to assist in reducing mortality for medical treatment.

**Methods:**

Electronic databases including PubMed, Web of Science, EMBASE, Cochrane Library, and Scopus were systematically searched till June 1, 2023. All human research were incorporated into the analysis, regardless of language, publication date, or geographical location. To pool the estimate, a mixed-effect model was used. The Newcastle–Ottawa Scale (NOS) was employed for assessing the quality of included studies that were included in the analysis.

**Results:**

In total, 22 studies with a total of 3655 severe pneumonia patients and 1107 cases (30.29%) of death were included in the current meta-analysis. Significant associations were found between age [5.76 years, 95% confidence interval [CI] (3.43, 8.09), *P* < 0.00001], male gender [odds ratio (OR) = 1.47, 95% CI (1.07, 2.02), *P* = 0.02], and risk of death from severe pneumonia. The comorbidity of neoplasm [OR = 3.37, 95% CI (1.07, 10.57), *P* = 0.04], besides the presence of complications such as diastolic hypotension [OR = 2.60, 95% CI (1.45, 4.67), *P* = 0.001], ALI/ARDS [OR = 3.63, 95% CI (1.78, 7.39), *P* = 0.0004], septic shock [OR = 9.43, 95% CI (4.39, 20.28), *P* < 0.00001], MOF [OR = 4.34, 95% CI (2.36, 7.95), *P* < 0.00001], acute kidney injury [OR = 2.45, 95% CI (1.14, 5.26), *P* = 0.02], and metabolic acidosis [OR = 5.88, 95% CI (1.51, 22.88), *P* = 0.01] were associated with significantly higher risk of death among patients with severe pneumonia. Those who died, compared with those who survived, differed on multiple biomarkers on admission including serum creatinine [Scr: + 67.77 mmol/L, 95% CI (47.21, 88.34), *P* < 0.00001], blood urea nitrogen [BUN: + 6.26 mmol/L, 95% CI (1.49, 11.03),* P* = 0.01], C-reactive protein [CRP: + 33.09 mg/L, 95% CI (3.01, 63.18), *P* = 0.03], leukopenia [OR = 2.63, 95% CI (1.34, 5.18), *P* = 0.005], sodium < 136 mEq/L [OR = 2.63, 95% CI (1.34, 5.18), *P* = 0.005], albumin [− 5.17 g/L, 95% CI (− 7.09, − 3.25), *P* < 0.00001], PaO_2_/FiO_2_ [− 55.05 mmHg, 95% CI (− 60.11, − 50.00), *P* < 0.00001], arterial blood PH [− 0.09, 95% CI (− 0.15, − 0.04), *P* = 0.0005], gram-negative microorganism [OR = 2.56, 95% CI (1.17, 5.62), *P* = 0.02], and multilobar or bilateral involvement [OR = 3.65, 95% CI (2.70, 4.93), *P* < 0.00001].

**Conclusions:**

Older age and male gender might face a greater risk of death in severe pneumonia individuals. The mortality of severe pneumonia may also be significantly impacted by complications such diastolic hypotension, ALI/ARDS, septic shock, MOF, acute kidney injury, and metabolic acidosis, as well as the comorbidity of neoplasm, and laboratory indicators involving Scr, BUN, CRP, leukopenia, sodium, albumin, PaO_2_/FiO_2_, arterial blood PH, gram-negative microorganism, and multilobar or bilateral involvement.

**Systematic review registration:**

PROSPERO Protocol Number: CRD 42023430684.

**Supplementary Information:**

The online version contains supplementary material available at 10.1186/s13643-024-02621-1.

## Introduction

Data from the 2019 Global Burden of Disease Study reported that lower respiratory infections were the fourth leading cause of mortality worldwide, accounting for over 2.49 million deaths, behind only neonatal disorders, ischemic heart disease, and stroke [1]. Severe pneumonia is a frequently common serious condition characterized by lower respiratory infection, with a high mortality, several complications, a poor prognosis, and a substantial economic burden [2]. Besides, it is a leading cause of ICU admission and infection-related death around the globe [3]. In the USA, pneumonia is to blame for 78% of infection-related deaths [4]. Despite the continuous advances in treatment over the past several decades, severe pneumonia has always been associated with a high mortality rate, ranging from 20% to more than 50% [5]. Therefore, it is crucial to investigate factors that contribute to the mortality of patients with severe pneumonia.

Identifying long-term mortality risk factors is critical for physicians to identify at-risk patients and for researchers to conduct interventional trials aiming at improving clinical outcomes [6]. Unreliable predictors may do more harm than benefit when used to guide clinical decisions [7]. For example, risky and aggressive therapies might be performed if the risk of poor outcomes is inaccurately characterized as high based on unreliable predictors. There are plenty of primary studies that attempt to discover prognostic factors for severe pneumonia, but frequently the results are inconsistent, the quality of the studies is inconsistent, and the predictive value of the majority of these potential prognostic factors has not been thoroughly assessed. Therefore, it is essential to identify, assess, and synthesize prognostic factor studies by applying systematic reviews and meta-analyses.

Systematic reviews and meta-analyses, which are key evidence synthesis methodologies, are widely employed in addressing varied healthcare concerns, and they are the foundation of evidence-based healthcare, providing evidence to support decision-making [8]. The majority of systematic reviews focus on summarizing the efficacy of interventions for a specific disease. Nonetheless, they are also essential for summarizing other evidence, such as the accuracy of screening and diagnostic tests, the causal association between risk factors and disease onset, and the prognostic ability of specific factors and biomarkers [9]. As a result, we sought to increase our understanding of severe pneumonia by conducting a systematic meta-analysis of published articles in order to thoroughly explain factors associated with mortality in hospitalized patients with severe pneumonia.

## Methods

### Protocol

This article has been reported in accordance with the Preferred Reporting Items for Systematic Reviews and Meta-Analyses (PRISMA) checklist [10] and registered in the PROSPERO database (CRD 42023430684).

### Data sources

By using PubMed, Web of Science, EMBASE, Cochrane Library, and Scopus from inception to June 2023 without regard to language, we carried out a retrospective, cross‐sectional systematic review. “severe pneumonia OR severe pulmonary inflammation OR severe pulmonary infection OR severe community acquired pneumonia OR severe hospital acquired pneumonia” (Title/Abstract) AND “mortality OR death OR died OR prognosis OR characteristics OR risk factors OR surviv* OR decease* OR fatal*” (All fields) were the search terms that we used. To ensure that we avoided ignoring any eligible studies, the references of meta-analyses or systematic review articles were additionally searched.

### Study selection

**Fig. 1:**
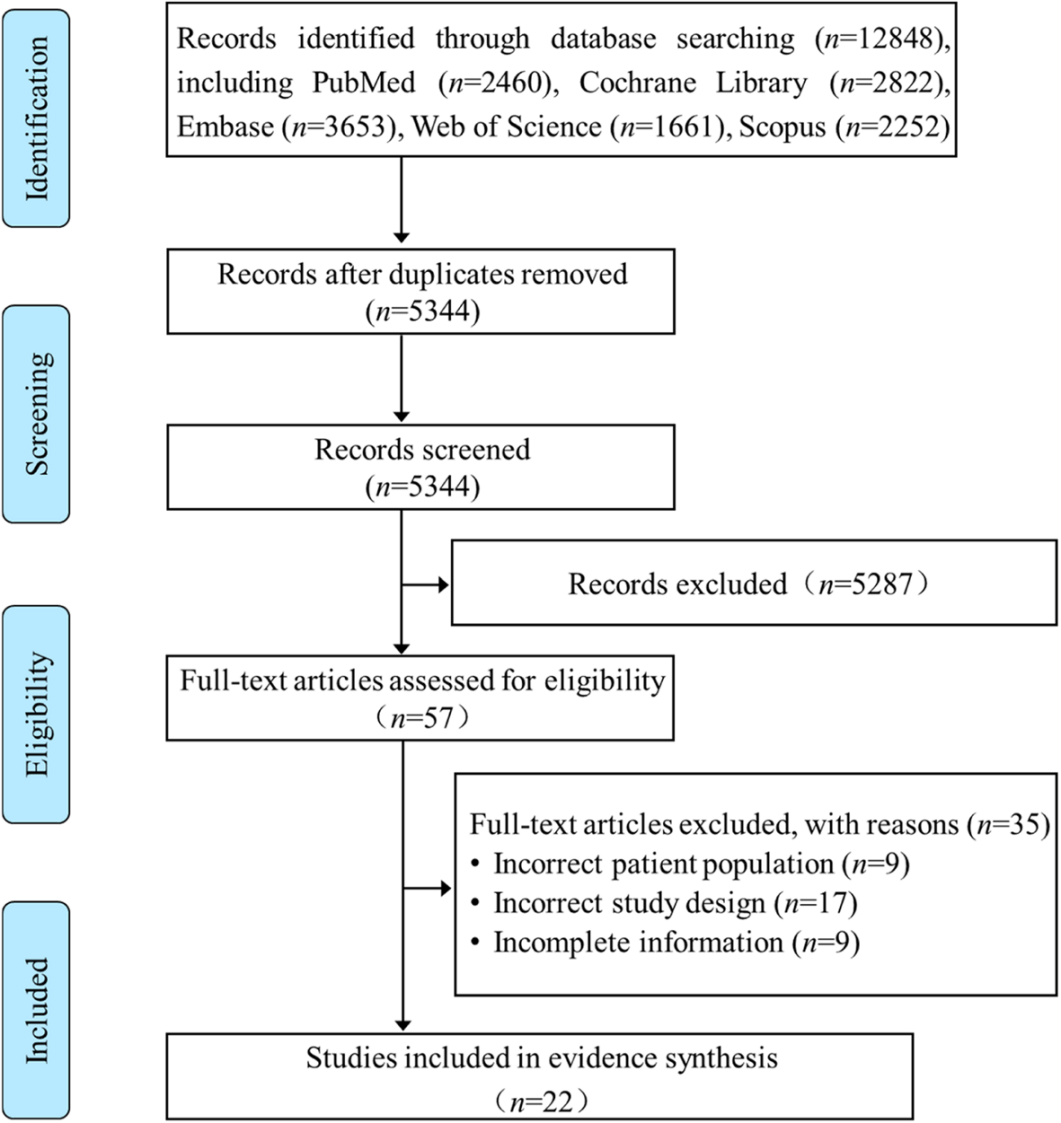
Flow diagram of the literature reviewing process and results

Two independent investigators reviewed the initial search results for relevant content with the titles and abstracts, and disagreements were solved by consensus. The full texts were reviewed for the eligibility criteria (Fig. 1).

The criteria for the inclusion of studies were as follows: (1) participants—patients with confirmed severe pneumonia; (2) design—primary studies with individual data for each mortality outcome group, i.e., survivors and non-survivors; (3) exposure variables—demographical characteristics, comorbidities, complications, clinical manifestations, laboratory results, or long-term prognosis outcomes; and (4) outcome—all patients observe to definitive hospital discharge or severe pneumonia mortality.

Noneligible publication types, such as duplicate publications, reviews, editorials, vitro or animal studies, case reports, family‐based studies, opinions, comments, responses, or other non-data driven article-types, or pediatric‐only cases were excluded. Only the article of superior quality was used into the analysis, in cases where multiple studies were published based on the identical patient sample and authored by the same individual. To enhance homogeneity, we selected for excluding studies that explicitly focused on fungal and viral pneumonia, such as severe H1N1, SARS, or COVID-19.

### Data extraction

The data extracted by two investigators independently from the included studies involved various aspects, including study design, the name of the first author, publication year, patient demographics (age and gender), sample size, exposure variables (risk factors), outcome variables (mortality risk), methods employed for exposure and outcome assessment, the primary adjusted risk estimate (expressed as hazard ratios, odds ratios, or relative risks) along with 95% confidence intervals (95% CI), and the adjusted confounding variables. Disagreements were resolved until consensus was reached by mutual discussion with a third reviewer.

### Quality assessment

Two independent reviewers used the Newcastle–Ottawa Scale (NOS) to evaluate the quality of included studies through the three criteria listed above: patient selection for the study, confounding variable adjustment, and outcome evaluation [11]. Each study may receive a maximum of 9 points based on this scale. Scores of 0 to 3, 4 to 6, and 7 to 9 were defined as low-, moderate-, and high-quality studies, respectively. Disagreements were resolved until consensus was reached by mutual discussion with a third reviewer.

### Statistical analysis

A meta-analysis was conducted using the program Review Manager (RevMan) (https://training.cochrane.org/online-learning/core-software-cochrane-reviews/revman) for comparing clinical features between patients with severe pneumonia who survived and those who did not. For continuous variables, whenever two or more studies reported a particular parameter, we estimated weighted mean differences and 95% CI in patients with severe pneumonia who survived vs. those who did not. We weighted the studies included in the meta-analysis using the generic inverse variance approach in RevMan. Additionally, RevMan was used to calculate measures of heterogeneity such as the χ^2^ and *I*^2^ statistics and the Tau^2^ statistic for random‐effects analysis [12]. When the statistical heterogeneity between studies was small (*P* ≥ 0.10, *I*^2^ ≤ 50%), use fixed effect model; on the contrary, if the statistical heterogeneity among the studies was large (*P* < 0.10, *I*^2^ > 50%), the random effect model was used and sensitivity analysis was performed to find the source of heterogeneity. If the heterogeneity was too large, further subgroup analysis was performed.

## Results

### Study search

A comprehensive review of papers published on or prior to June 1, 2023, yielded a total of 12,848 topic-related articles. 7504 duplicates and 5287 studies that did not meet the qualifying criteria were eliminated, resulting in 57 papers that remained for further review. Papers for 35 were eliminated due to the following three primary factors: incorrect patient population, incorrect study design, and insufficient information. Ultimately, we got 22 studies that met all of the inclusion criteria (Fig. 1).

### Study characteristics

A total of 3655 participants were enrolled in the investigations. Out of a total of 22 studies, three were conducted in mainland China and three in Taiwan, China. Additionally, three studies were carried out in Singapore, while two studies each were conducted in France, the USA, Tunisia, and Spain. Furthermore, one study each was performed in the UK, Egypt, Australia, Russia, and South Africa. The enrolled patient sample size varied from 8 to 815 individuals. Overall, 1107 patients died (30.3%). Out of the total 808 participants, 501 individuals (62.0%) were identified as male gender. Additionally, the average age of the patients enrolled was 69.8 years. The main characteristics of the included studies are reported in Table 1.

**Table 1:** Characteristics of studies included in the systematic review and meta-analysis

Study	Publication year	Sample size (survivor/non-survivor)	Study design	Country
Pallares et al. [13]	1995	504 (364/140)	Prospective	Spain
Potgieter et al. [14]	1996	58 (46/12)	Prospective	The USA
El-Ebiary et al. [15]	1997	84 (59/25)	Prospective	Spain
Hirani et al. [16]	1997	57 (24/33)	Retrospective	UK
Sikka et al. [17]	2000	104 (47/57)	Prospective	The USA
Feldman et al. [18]	2001	182 (106/76)	Prospective	South Africa
Paganin et al. [19]	2004	112 (64/48)	Prospective	France
Wilson et al. [20]	2005	96 (65/31)	Prospective	Australia
Poulose V et al. [21]	2008	80 (56/24)	Retrospective	Singapore
Ong et al. [22]	2009	8 (3/5)	Retrospective	Singapore
Lee et al. [23]	2010	112 (55/57)	Retrospective	Taiwan, China
Phua et al. [24]	2010	74 (45/29)	Retrospective	Singapore
Belkhouja et al. [25]	2012	132 (99/33)	Retrospective	Tunisia
Georges et al. [26]	2013	317 (201/116)	Retrospective and prospective	France
Fekih et al. [27]	2014	209 (149/60)	Prospective	Tunisia
Chien et al. [28]	2015	40 (25/15)	Prospective	Taiwan, China
Sakharov et al. [29]	2020	60 (30/30)	Retrospective	Russia
Abdelaziz et al. [30]	2021	100 (59/41)	Prospective	Egypt
Tseng et al. [31]	2021	815 (678/137)	Retrospective	Taiwan, China
Geng et al. [32]	2022	119 (83/36)	Retrospective	China
Zhang K et al. [33]	2023	240 (183/57)	Multicenter prospective	China
Zhang C et al. [34]	2023	152 (107/45)	NA	China

### Demographical characteristics

**Fig. 2:**
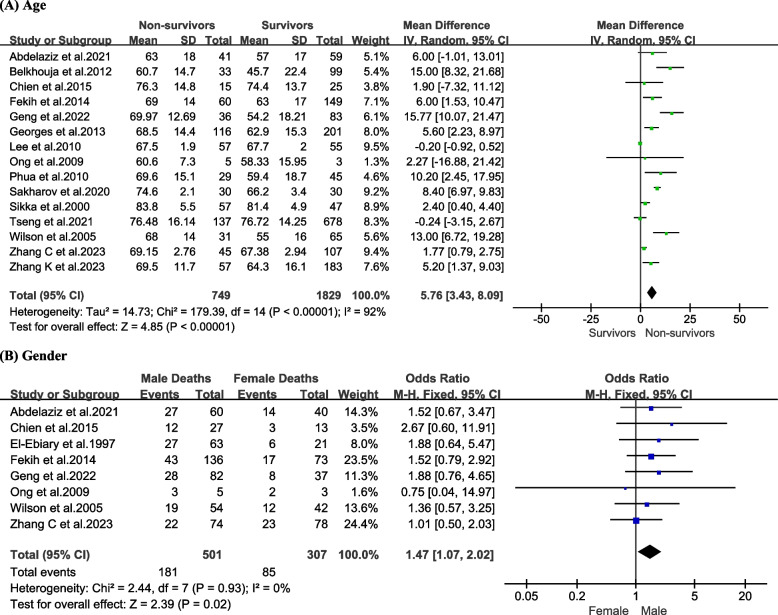
Forest plots demonstrating the association between severe pneumonia mortality and the presence of age (**A**) and gender (**B**). Sizes of data markers indicate weight of studies. CI, confidence intervals; df, degrees of freedom; IV, inverse variance

An increased risk of death was found to be associated with older age [Table 2 and Fig. 2A, mean difference 5.76, 95% CI (3.43, 8.09), *P* < 0.00001]. Furthermore, male gender was associated with a higher mortality risk [Table 3 and Fig. 2B; odds ratio [OR] = 1.47, 95% CI (1.07, 2.02), *P* = 0.02].

**Table 2:** Meta-analysis results of continuous variable

Variable	No. of studies	Mean difference (95% CI) non-survivor‐survivor	Meta‐analysis *P* value
Demographical characteristics			
Age (years)	15	5.76 (3.43, 8.09)	< 0.00001*
Clinical manifestations			
Respiratory rate (breaths/min)	3	2.14 (− 1.23, 5.51)	0.21
Heart rate (beats/min)	3	1.37 (− 6.03, 8.78)	0.72
Body temperature (°C)	2	− 0.35 (− 0.71, 0.01)	0.05
MAP (mmHg)	2	− 8.24 (− 23.80, 7.32)	0.3
Urine output (mL)	2	− 355.79 (− 834.27, 122.70)	0.15
Laboratory results			
WBC count (× 10^9^/L)	5	− 0.04 (− 5.34, 5.26)	0.99
Platelet count (cells/mm^3^)	4	− 8.79 (− 19.23, 1.66)	0.10
CRP (mg/L)	3	33.09 (3.01, 63.18)	0.03*
Hemoglobin (g/L)	3	− 2.86 (− 10.78, 5.06)	0.48
Albumin (g/L)	4	− 5.17 (− 7.09, − 3.25)	< 0.00001*
AST (U/L)	2	25.80 (− 48.11, 99.72)	0.49
Scr (mmol/L)	4	67.77 (47.21, 88.34)	< 0.00001*
BUN (mmol/L)	4	6.26 (1.49, 11.03)	0.01*
Potassium (mmol/L)	2	0.33 (− 0.22, 0.88)	0.24
PaO_2_/FiO_2_	5	− 55.05 (− 60.11, − 50.00)	< 0.00001*
Arterial blood PH	2	− 0.09 (− 0.15, − 0.04)	0.0005*
Long-term prognosis outcomes			
Length of hospital stay (days)	4	0.49 (− 2.09, 3.08)	0.71
Length of ICU stay (days)	2	− 0.09 (− 8.67, 8.48)	0.98

Abbreviations: *WBC*, white blood cell; *AST*, glutamic oxaloacetic transaminase; *Scr*, serum creatinine; *BUN*, blood urea nitrogen; *CRP*, C-reactive protein; *MAP*, mean arterial pressure

^*^P < 0.05

**Table 3:** Meta-analysis results of two categorical variables

Variable	No. of studies	Survivors		Non-survivors		OR (95% CI)	Meta‐analysis *P* value
		Total (survivors)	No. with risk factor (%)	Total (non-survivors)	No. with risk factor (%)		
Demographical characteristics							
Male gender	8	501	320 (63.9)	266	181 (68.0)	1.47 (1.07, 2.02)	0.02*
Comorbidities							
COPD	7	568	135 (23.8)	236	57 (24.2)	0.91 (0.52, 1.58)	0.73
Hypertension	5	494	194 (39.3)	207	70 (33.8)	0.78 (0.55, 1.10)	0.16
Neoplasm	5	375	17 (4.5)	177	27 (15.3)	3.37 (1.07, 10.57)	0.04*
Diabetes mellitus	11	1223	236 (19.3)	513	97 (18.9)	1.03 (0.78, 1.35)	0.85
Complications							
Diastolic hypotension	2	131	31 (23.7)	107	43 (40.2)	2.60 (1.45, 4.67)	0.001*
Respiratory failure	3	176	23 (13.1)	98	30 (30.6)	1.37 (0.59, 3.18)	0.47
Acute confusion	2	57	11 (19.3)	23	8 (34.8)	1.69 (0.53, 5.43)	0.37
Pleural effusion	2	104	31 (29.8)	54	18 (33.3)	0.95 (0.14, 6.45)	0.95
ALI/ARDS	2	129	34 (26.4)	63	39 (61.9)	3.63 (1.78, 7.39)	0.0004*
Bacteremia	3	167	58 (34.7)	67	41 (61.2)	0.91 (0.40, 2.05)	0.82
Septic shock	7	509	122 (24.0)	247	181 (73.3)	9.43 (4.39, 20.28)	< 0.00001*
MOF	2	182	33 (18.1)	69	34 (49.3)	4.34 (2.36, 7.95)	< 0.00001*
Acute kidney injury	6	370	58 (15.7)	199	60 (30.2)	2.45 (1.14, 5.26)	0.02*
Metabolic acidosis	2	129	32 (24.8)	79	53 (67.1)	5.88 (1.51, 22.88)	0.01*
Laboratory results							
Leukopenia	2	266	19 (7.1)	147	27 (18.4)	2.63 (1.34, 5.18)	0.005*
Multilobar or bilateral involvement	6	718	231 (32.2)	356	208 (58.4)	3.65 (2.70, 4.93)	< 0.00001*
Bacterial mixed infection	3	472	38 (8.1)	191	40 (20.9)	2.18 (0.72, 6.55)	0.17
Positive blood culture	2	89	21 (23.6)	63	26 (41.3)	1.84 (0.52, 6.49)	0.35
Sputum cont culture growth	2	742	370 (49.9)	185	105 (56.8)	1.07 (0.76, 1.51)	0.70
Gram-negative microorganism	2	108	41 (38.0)	51	27 (52.9)	2.56 (1.17, 5.62)	0.02*
Gram-positive microorganism	2	108	10 (9.3)	51	8 (15.7)	1.76 (0.64, 4.81)	0.27
Sodium < 136 mEq/L	2	123	31 (25.2)	73	36 (49.3)	2.63 (1.34, 5.18)	0.005*

Abbreviations: *COPD*, chronic obstructive pulmonary disease; *OR*, odds ratio; *MOF*, multiple organ failure; *ALI/ARDS*, acute lung injury/acute respiratory distress syndrome

^*^P < 0.05

### Comorbidities

**Fig. 3:**
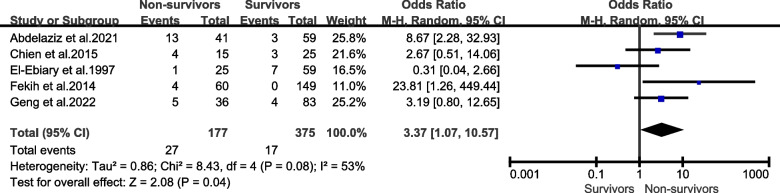
Forest plots demonstrating the association between severe pneumonia mortality and the presence of neoplasm

In our conducted meta-analysis, the comorbidity of neoplasm were identified as being strongly associated with an increased risk of mortality attributed to severe pneumonia [Fig. 3, OR = 3.37, 95% CI (1.07, 10.57), *P* = 0.04] (summarized in Table 3).

No significant association was observed between the risk of death from severe pneumonia and comorbidities including COPD [Fig. S2A, OR = 0.91, 95% CI (0.52, 1.58), *P* = 0.73], hypertension [Fig. S2B, OR = 0.78, 95% CI (0.55, 1.10), *P* = 0.16], and diabetes mellitus [Fig. S2C, OR = 1.03, 95% CI (0.78, 1.35), *P* = 0.85].

### Complications

Among the whole patient population, bacteremia emerged as the most prevalent complication among patients that died with an incidence of 42.3%, followed by metabolic acidosis (40.9%), septic shock (40.1%), acute kidney injury (39.7%), ALI/ARDS (38.0%), and multiple organ failure (MOF, 26.7%).

**Fig. 4:**
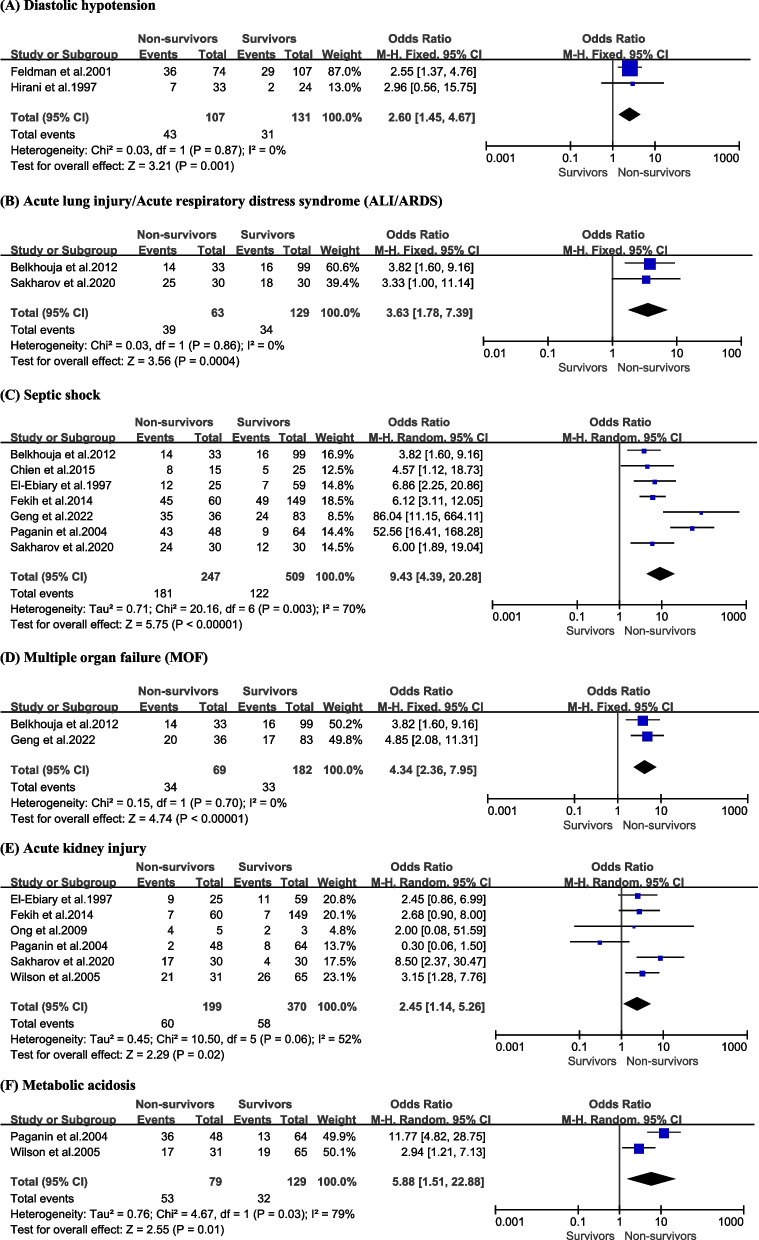
Forest plots demonstrating the association between severe pneumonia mortality and the presence of diastolic hypotension (**A**), acute lung injury/acute respiratory distress syndrome (**B**), septic shock (**C**), multiple organ failure (**D**), acute kidney injury (**E**), and metabolic acidosis (**F**)

In our conducted meta-analysis, several complications were identified as being strongly associated with an increased risk of mortality attributed to severe pneumonia (summarized in Table 3), including diastolic hypotension [Fig. 4A, OR = 2.60, 95% CI (1.45, 4.67), *P* = 0.001], ALI/ARDS [Fig. 4B, OR = 3.63, 95% CI (1.78, 7.39), *P* = 0.0004], septic shock [Fig. 4C, OR = 9.43, 95% CI (4.39, 20.28), *P* < 0.00001], MOF [Fig. 4D, OR = 4.34, 95% CI (2.36, 7.95), *P* < 0.00001], acute kidney injury [Fig. 4E, OR = 2.45, 95% CI (1.14, 5.26), *P* = 0.02], and metabolic acidosis [Fig. 4F, OR = 5.88, 95% CI (1.51, 22.88), *P* = 0.01].

No significant association was observed between the risk of death from severe pneumonia and complications including respiratory failure [Fig. S3A, OR = 1.37, 95% CI (0.59, 3.18), *P* = 0.47], acute confusion [Fig. S3B, OR = 1.69, 95% CI (0.53, 5.43), *P* = 0.37], pleural effusion [Fig. S3C, OR = 0.95, 95% CI (0.14, 6.45), *P* = 0.95] and bacteremia [Fig. S3D, OR =0.91, 95% CI (0.40, 2.05), P=0.82].

### Clinical manifestations

There was no statistically significant association observed between mortality and the presence of respiratory rate [Fig. S1A, + 2.14 breaths/min, 95% CI (− 1.23, 5.51),* P* = 0.21], heart rate [Fig. S1B, + 1.37 beats/min, 95% CI (− 6.03, 8.78), *P* = 0.72], body temperature [Fig. S1C, − 0.35 °C, 95% CI (− 0.71, 0.01), *P* = 0.05], mean arterial pressure [Fig. S1D, MAP: − 8.24 mmHg, 95% CI (− 23.80, 7.32), *P* = 0.3], or urine output [Fig. S1E, − 355.79 mL, 95% CI (− 834.27, 122.70), *P* = 0.15] in severe pneumonia patients (Table 2).

### Laboratory results

**Fig. 5:**
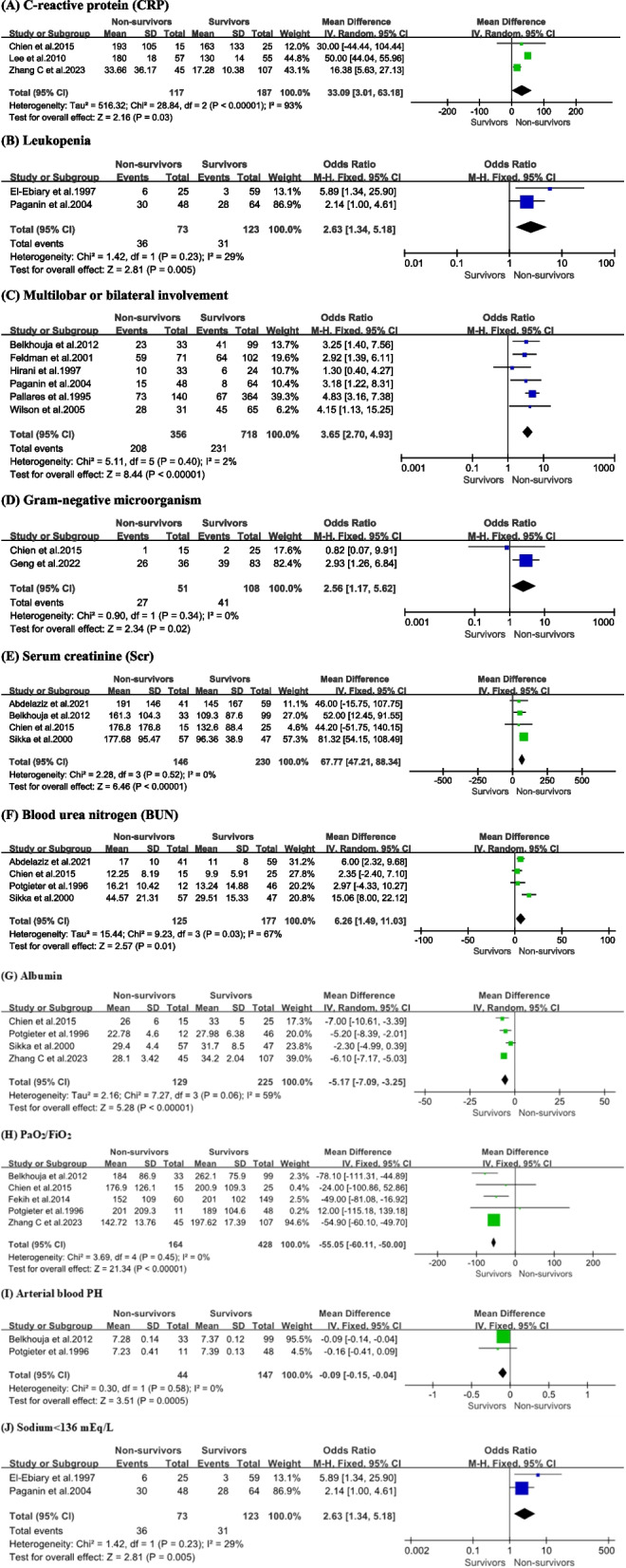
Forest plots demonstrating the association between severe pneumonia mortality and the presence of C-reactive protein (**A**), leukopenia (**B**), multilobar or bilateral involvement (**C**), gram-negative microorganism (**D**), serum creatinine (**E**), blood urea nitrogen (**F**), albumin (**G**), PaO_2_/FiO_2_ (**H**), arterial blood PH (**I**), and sodium < 136 mEq/L (**J**)

The relationship between frequent laboratory results and mortality was investigated (Tables 2 and 3) and Fig. 5, S4). The non-survivor group had higher levels of several severe pneumonia biomarkers. The non-survival and survival groups had varied levels of inflammatory factors, with the former displaying a higher level of C-reactive protein [CRP: Fig. 5A, + 33.09 mg/L, 95% CI (3.01, 63.18), *P* = 0.03]. Leukopenia is a medical disorder characterized by white blood cells count below 4 × 10^9^/L, and it is notably more frequent among individuals who do not survive [Fig. 5B, OR = 2.63, 95% CI (1.34, 5.18), *P* = 0.005]. Multilobar or bilateral involvement [Fig. 5C, OR = 3.65, 95% CI (2.70, 4.93), *P* < 0.00001] was consistently higher among those patients who died. The non-survival group exhibited greater amounts of gram-negative microorganisms than the survival group [Fig. 5D, OR = 2.56, 95% CI (1.17, 5.62), *P* = 0.02]. This study findings indicated that non-survivors had significantly higher levels of serum creatinine [Scr: Fig. 5E, + 67.77 mmol/L, 95% CI (47.21, 88.34), *P* < 0.00001] and blood urea nitrogen [BUN: Fig. 5F, + 6.26 mmol/L, 95% CI (1.49, 11.03),* P* = 0.01], suggesting a correlation between poorer renal function on hospital admission and greater mortality. The albumin [Fig. 5G, − 5.17 g/L, 95% CI (− 7.09, − 3.25), *P* < 0.00001], PaO_2_/FiO_2_ [Fig. 5H, − 55.05 mmHg, 95% CI (− 60.11, − 50.00), *P* < 0.00001], and arterial blood PH [Fig. 5I, − 0.09, 95% CI (− 0.15, − 0.04), *P* = 0.0005] tended to be lower in severe pneumonia patients who did not survive. Similarly, patients with severe pneumonia who died had higher percentages of blood sodium below 136 mEq/L [Fig. 5J, OR = 2.63, 95% CI (1.34, 5.18), *P* = 0.005].

We found that some laboratory results showed no statistically significant differences between survivors and non-survivors, including bacterial mixed infection [Fig. S4A, OR = 2.18, 95% CI (0.72, 6.55), *P* = 0.17], positive blood culture [Fig. S4B, OR = 1.84, 95% CI (0.52, 6.49), *P* = 0.35], sputum cont culture growth (Fig. S4C, OR = 1.07, 95% CI (0.76, 1.51), *P* = 0.70), gram-positive microorganism [Fig. S4D, OR = 1.76, 95% CI (0.64, 4.81), *P* = 0.27], WBC count [Fig. S4E, − 0.04 × 10^9^/L, 95% CI (− 5.34, 5.26), *P* = 0.99], platelet count [Fig. S4F, − 8.79 μg/L, 95% CI (− 19.23, 1.66), *P* = 0.1], hemoglobin [Fig. S4G, − 2.86 g/L, 95% CI (− 10.78, 5.06), *P* = 0.48], glutamic oxaloacetic transaminase [AST: Fig. S4H, + 25.80 U/L, 95% CI (− 48.11, 99.72), *P* = 0.49], and potassium [Fig. S4I, + 0.33 mmol/L, 95% CI (− 0.22, 0.88), *P* = 0.24].

### Long-term prognosis outcomes

The length of hospital stay [Fig. S5A, 0.49 d, 95% CI (− 2.09, 3.08), *P* = 0.71] and length of ICU stay [Fig. S5B, − 0.09 d, 95% CI (− 8.67, 8.48), *P* = 0.98] were not related with an increased risk of death in patients with severe pneumonia.

### Heterogeneity analysis

There were statistically significant differences between survivors and non-survivors in severe pneumonia on the basis of age, gender, complications with diastolic hypotension, ALI/ARDS, septic shock, MOF, acute kidney injury, metabolic acidosis, the comorbidity of a neoplasm, and laboratory tests such as Scr, BUN, CRP, leukopenia, sodium, albumin, PaO_2_/FiO_2_, arterial blood PH, gram-negative microorganism, and multilobar or bilateral involvement. However, there was large heterogeneity in age, septic shock, acute kidney injury, metabolic acidosis, neoplasm, BUN, CRP, and albumin (*I*^2^ > 50%). Therefore, we conducted an analysis to determine the source of this heterogeneity.

The mortality of severe pneumonia was significantly influenced by age in our study, with a high degree of heterogeneity (*P* < 0.00001, *I*^2^ = 92%). According to studies included in our article, different countries exhibit variations in their medical settings, with intensive care unit admission potentially serving as an indicator of the severity of a disease. Within subgroups, factors including sex ratio, sample size, and study design were evaluated. The results of our study indicated that there was substantial variation among each subgroup, indicating that the factors in consideration did not make a substantial contribution to the observed heterogeneity (Table 4). As a result, we formulated the hypothesis that intervention measures, treatment duration, and additional variables might exert an influence. However, a thorough comparison taking these aspects into account was not acquired due to the small amount of data available in the original literature review.

**Table 4:** Subgroup analysis for the association between age and mortality risk of severe pneumonia

Subgroup variables	No. of studies	OR (95% CI)	Measure of heterogeneity	
			*P*	*I*^2^
Geographic region		5.30 (2.96, 7.65)	< 0.00001	0.92
Africa	3	8.75 (3.13, 14.38)	0.002	0.62
Asia	6	2.92 (0.51, 5.33)	0.02	0.88
Europe	2	7.43 (4.82, 10.04)	< 0.00001	0.56
North America	2	4.69 (− 0.89, 10.27)	0.10	0.45
Study design		6.36 (3.41, 9.31)	< 0.0001	0.96
Retrospective	8	7.00 (2.52, 11.47)	0.0002	0.58
Prospective	6	5.42 (2.47, 8.37)	0.003	0.93
ICU admission		6.36 (3.41, 9.31)	< 0.0001	0.73
ICU	7	5.86 (1.20, 10.51)	< 0.0001	0.96
Not ICU	8	6.80 (3.40, 10.21)	0.01	0.93
Sex ratio (male/female)		6.12 (3.10, 9.15)	< 0.0001	0.93
≤ 2	7	7.21 (2.62, 11.80)	0.002	0.83
> 2	7	5.10 (0.75, 9.45)	0.02	0.96
Sample size		6.12 (3.10, 9.15)	< 0.0001	0.93
≤ 100	5	5.79 (0.43, 11.15)	0.03	0.80
100 ~ 200	5	8.37 (5.09, 11.65)	< 0.00001	0.70
> 200	4	3.31 (− 0.42, 7.03)	0.08	0.86

The statistical results of septic shock (*P* < 0.00001, *I*^2^ = 70%) revealed significant heterogeneity. By systematically removing individual studies, we identified that the elimination of Paganin et al. [19] resulted in non-significant heterogeneity (*P* < 0.00001, *I*^2^ = 41%), suggesting that this particular study was a major source of heterogeneity in relation to septic shock. Furthermore, this particular study also significantly in acute kidney injury, the statistical results changed from (*P* = 0.02, *I*^2^ = 52%) to (*P* < 0.00001, *I*^2^ = 0%) when removed the study of Paganin et al. [19]. Analysis of the original literature revealed that patients included in this study underwent mechanical ventilation therapy during early admission, which may account for the observed heterogeneity. Additionally, when El-Ebiary et al. [15] article removed, the neoplasm statistics transformed from (*P* = 0.04,* I*^2^ = 53%) to (*P* < 0.00001, *I*^2^ = 0%). Further analysis indicated that patients received antibiotic treatment prior to admission—a significant factor contributing to neoplasm heterogeneity. The remaining outcome indicators of metabolic acidosis, BUN, CRP, and albumin were reported in less than five studies, so the heterogeneity of the meta-analysis was high.

### Quality assessment

The Newcastle–Ottawa score of the included studies was 7 ~ 9, and the quality of all articles was evaluated as high (Table 5).

**Table 5:** Quality assessment of the eligible studies with Newcastle–Ottawa Scale

Study	Selection (4)				Comparability (2)		Outcome (3)			Total
	Representativeness	Selection of non-exposed	Ascertainment of exposure	Outcome not present at start	Study control for age and sex	Comparability on other risk factors (controlled for ≥ 2 variables)	Assessment of outcome	Long enough follow-up (median ≥ 1 year)	Adequacy (completeness) of follow-up	
Pallares et al. [13]	1	1	1	1	0	1	1	1	1	8
Potgieter et al. [14]	1	1	1	1	0	1	1	1	1	8
El-Ebiary et al. [15]	1	1	1	1	0	1	1	1	1	8
Hirani et al. [16]	1	1	1	1	0	1	1	1	1	8
Sikka et al. [17]	1	1	1	1	0	1	1	1	1	8
Feldman et al. [18]	1	1	1	1	0	1	1	1	1	8
Paganin et al. [19]	1	1	1	1	0	1	1	1	1	8
Wilson et al. [20]	1	1	1	1	1	1	1	1	1	9
Poulose V et al. [21]	1	1	1	1	0	0	1	1	1	7
Ong et al. [22]	1	1	1	1	1	1	1	1	1	9
Lee et al. [23]	1	1	1	1	1	1	1	1	1	9
Phua et al. [24]	1	1	1	1	0	1	1	1	1	8
Belkhouja et al. [25]	1	1	1	1	0	1	1	1	1	8
Georges et al. [26]	1	1	1	1	0	1	1	1	1	8
Fekih et al. [27]	1	1	1	1	1	1	1	1	1	9
Chien et al. [28]	1	1	1	1	1	1	1	1	1	9
Sakharov et al. [29]	1	1	1	1	0	1	1	1	1	8
Abdelaziz et al. [30]	1	1	1	1	1	1	1	1	1	9
Tseng et al. [31]	1	1	1	1	1	1	1	1	1	9
Geng et al. [32]	1	1	1	1	1	1	1	1	1	9
Zhang K et al. [33]	1	1	1	1	1	1	1	1	1	9
Zhang C et al. [34]	1	1	1	1	1	1	1	1	1	9

## Discussion

This study presents a systematic review and meta-analysis of 22 published articles, covering a total of 3655 patients. It aims to offer a comprehensive analysis of various factors, including demographical characteristics, comorbidities, complications, clinical manifestations, laboratory results, and long-term prognosis outcomes, that are associated with mortality in cases of severe pneumonia. Notably, this study is the first of that sort to provide such a comprehensive analysis.

### Principal findings

The primary finding of our study revealed mortality of 30.3% for severe pneumonia, a rate that is consistent with the results of previous research [35]. As individuals age, the immune system experiences a range of alterations, ultimately resulting in a decreased capacity to effectively initiate a cellular response to combat infections [36]. Polymorphonuclear leukocytes in the elderly exhibit weakened chemotactic capacity, as well as lower microbe uptake, and antigen processing ability of macrophages [37]. Similarly, our findings revealed a substantial connection between old age and severe pneumonia mortality, which in accordance with previous research [38]. One probable explanation could be age-related chronic medical issues and/or a reduced immune level [39]. Our meta-analysis additionally showed that male gender appeared to be a risk factor for severe pneumonia mortality. Sex differences in the adaptive and innate immune systems have already been identified, which may account for the women’s advantage in severe pneumonia [40, 41]. In the adaptive immune system, men have fewer CD8 + T cells [42], fewer CD4 + T cells [43], and less B cell generation than women [43].

Severe pneumonia is a severe respiratory disease accompanied by other comorbidities or complications in the field of clinical care. Based on our comprehensive analysis, it was found that several factors, namely diastolic hypotension, ALI/ARDS, septic shock, MOF, acute kidney injury, and metabolic acidosis, as well as the comorbidity of neoplasm, were identified as major risk factors associated with death in individuals with severe pneumonia. The assessment and administration of intravascular volume status hold significant importance in critically ill individuals. Hypotension is frequently attributed to low blood volume resulting from either bleeding or fluid transfer during systemic inflammation [44]. Additionally, the study we did revealed a significant correlation between diastolic hypotension and mortality resulting from severe pneumonia. Immunity runs through the development of neoplasm [45], as is the occurrence of severe pneumonia. Moreover, severe pneumonia often presents with pulmonary and extra-pulmonary consequences, such as ALI/ARDS, septic shock, and MOF. The primary pathophysiological characteristic of ALI/ARDS is the presence of an inflammatory storm. A growing number of evidence indicates that immune cells and the cytokines they generate play a crucial role in the pathophysiology of ALI/ARDS [39]. This could perhaps explain the increased mortality observed in individuals with severe pneumonia who also present with ALI/ARDS. In addition, a retrospective clinical investigation using a sample size of 710 patients indicated that the mortality rate for those with severe pneumonia and septic shock was greater compared to those without septic shock [46]. This is not unexpected considering that shock is an accepted main severity criterion for community-acquired pneumonia (CAP) and is associated with clinical failure [47]. MOF is a pathological condition resulting from an imbalanced inflammatory response, and it is the primary cause of death in critically patients [48]. This observation supports our discovery that the presence of MOF significantly enhances the risk of mortality due to severe pneumonia. Acute inflammation has been observed to negatively affect endothelial functioning, resulting in an imbalance between vasodilatory and vasoconstrictive processes. This imbalance has the potential to contribute to the development of MOF [49]. Similarly, our study revealed that individuals affect with severe pneumonia in combination with neoplasm exhibited a higher incidence of mortality. Numerous investigations have indicated the existence of a complex interaction between the pulmonary and renal systems [50]. The lung and kidney are vital organs responsible for maintaining acid–base balance and fluid balance. Consequently, any damage to the kidney can significantly impact the lung by disrupting the normal balance of acid–base levels and fluid distribution. Furthermore, the kidney can also contribute to the development and regulation of lung diseases through the production or clearance of mediators. This interplay between the lung and kidney underscores their interdependence and mutual influence on overall physiological function [51]. According to a study, it was shown that patients who experienced both acute renal damage and pneumonia exhibited a higher possibility of mortality compared to individuals who either had acute kidney injury or pneumonia separately [52]. Our study also showed that the existence of acute kidney damage was identified as risk factor for increased mortality in severe pneumonia cases. This link may be attributed to the correlation between the lung microbiota of patients with severe pneumonia and the occurrence of kidney injury [53]. Due to the inherent characteristics of critical disease, patients admitted to the intensive care unit (ICU) may experience a range of acid–base variations. Sepsis, renal failure, and impaired respiratory function all disrupt the body’s capacity to regulate pH levels and sustain homeostasis. Increased mortality rates have been observed in relation to alterations in blood pH levels [54]. Not surprisingly, individuals with severe pneumonia who also exhibited metabolic acidosis experience a greater death rate.

The ability to classify patients at an earlier stage of the illness process could be of great value for promoting earlier referrals and potentially enhancing patient outcomes. This, in turn, has implications for decision-making at the individual, provider, and system levels [55]. To this end, we included laboratory results in our analysis. Various inflammatory factors produced by the inflammatory storm can cause systemic immune damage in severe pneumonia patients [56]. In the current study, it was revealed that individuals belonging to the non-survival group exhibited a higher tendency of CRP levels and leukopenia. Furthermore, it was observed that non-survivors exhibited electrolyte disruption, as well as impaired kidney and liver function, upon admission, as indicated by the levels of albumin, Scr, BUN, and sodium, in comparison to survivors. Mortality was observed to have a correlation with reduced PaO_2_/FiO_2_ and arterial blood pH levels, indicating a potential presence of respiratory failure and acid–base imbalance among these severe pneumonia individuals. The presence of a large concentration of gram-negative microorganisms and multilobar or bilateral involvement have been found to be strongly associated with a higher risk of mortality. Overall, these findings indicate that conducting an initial laboratory evaluation is crucial for categorizing the risk level of patients with severe pneumonia. Patients who exhibit indicators of end-organ dysfunction, inflammation, respiratory dysfunction, or acid–base imbalance are more likely to experience an unfavorable outcome.

It is noteworthy to acknowledge that our study revealed the absence of significant correlation between the indicators and manifestations of respiratory rate, heart rate, body temperature, MAP, urine output, as well as the length of hospital stay and ICU stay, and the mortality rate among individuals diagnosed with severe pneumonia.

### Limitations

Despite the inclusion of pooled estimates from 22 studies conducted in 12 different geographical regions, our study is subject to several limitations. First, a notable degree of variety is observed. The observed phenomenon may be ascribed to the substantial disparity in sample sizes between research, ranging from 8 to 815 patients, as well as the variation in study methodologies. Second, it should be noted that many studies incorporated into the analysis had a limited sample size, perhaps hindering the ability to identify any important variables that contribute to mortality rates in cases of severe pneumonia. Third, there existed considerable heterogeneity among factors that exhibit statistically significant associations with mortality outcomes of severe pneumonia. These factors comprise age, septic shock, acute kidney injury, metabolic acidosis, neoplasm, BUN, CRP, and albumin. Though, a heterogeneity analysis was performed in an effort to identify the source of heterogeneity, as a result of the small number of studies and the limitations associated with acquiring data from the primary study, we were unable to completely interpret in some indicators. Thus, further high-quality research is required to confirm our results. Fourth, all of the included studies were non-randomized controlled trials, and there could be a number of confounding factors likely remained that affecting the external validity of the findings. Thus, further analyses with studies considering more confounder effects and more publications included would be necessary. In addition to the indicators presented in our systematic review, future studies ought to probe into more factors that may influence mortality of severe pneumonia. Fifth, although we conducted as thorough and systematic search of published literature as possible, the studies that met the eligibility criteria only covered 12 countries, with Asia being the largest region, so we must admit that the study population representation of our results may be underrepresented, and that it is applicable in different regions of the world needs to be verified by further trials. Ultimately, the number of literatures related to severe pneumonia is continuously expanding, with new information and research articles being published on a daily basis. Consequently, it is important to acknowledge that our study does not provide a comprehensive analysis of the topic.

## Conclusion

Our study revealed consistent and statistically significant associations between various factors and the fatal outcome of severe pneumonia. These factors include male gender, older age, the comorbidity of neoplasm, complications including diastolic hypotension, ALI/ARDS, septic shock, MOF, acute kidney injury, metabolic acidosis, and laboratory results including Scr, BUN, CRP, leukopenia, sodium, albumin, PaO_2_/FiO_2_, arterial blood PH, gram-negative microorganism, and multilobar or bilateral involvement. In order to decrease the risk of mortality of patients with severe pneumonia, it is essential to systematically develop and conduct public health programs specifically targeting those who are at risk.

### Supplementary Information

Additional file 1. Supplementary figures.

## Data Availability

The original contributions presented in this study are included in the article/supplement. For further inquiries, please contact the corresponding author.

## Abbreviations

NOS: The Newcastle–Ottawa Scale
RevMan: Review Manager
COPD: Chronic obstructive pulmonary disease
OR: Odds ratio
MOF: Multiple organ failure
ALI/ARDS: Acute lung injury/acute respiratory distress syndrome
WBC: White blood cell
AST: Glutamic oxaloacetic transaminase
Scr: Serum creatinine
BUN: Blood urea nitrogen
CRP: C-reactive protein
MAP: Mean arterial pressure
CAP: Community-acquired pneumonia
ICU: Intensive care unit
