# Supplementary material for: Predictors of mortality in severe pneumonia patients: a systematic review and meta-analysis
Source: Syst Rev. 2024 Aug 5;13:210. doi: 10.1186/s13643-024-02621-1 (PMC11302088; doi:10.1186/s13643-024-02621-1)
Supplement: Supplementary file 1 — Additional file 1. Supplementary figures. [file 13643_2024_2621_MOESM1_ESM.docx]

**Supplementary Online Content**

**Supplemental Figure 1:** Association between the clinical manifestations and severe pneumonia mortality.

**Supplemental Figure 2:** Association between the comorbidities and severe pneumonia mortality.

**Supplemental Figure 3:** Association between the complications and severe pneumonia mortality.

**Supplemental Figure 4:** Association between the laboratory results and severe pneumonia mortality.

**Supplemental Figure 5:** Association between the long-term prognosis outcomes and severe pneumonia mortality.

**Supplemental Figure 1:** Association between the clinical manifestations and severe pneumonia mortality.

**(A) Respiratory rate**

**
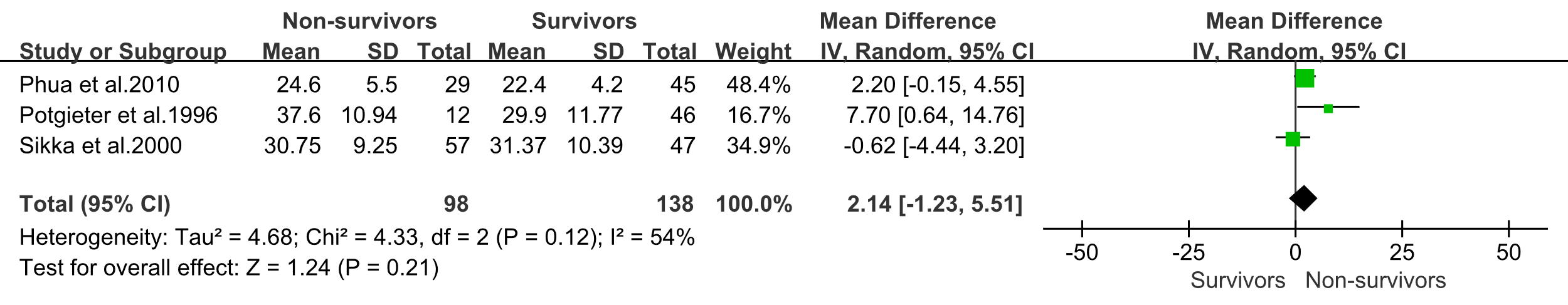
**

**(B) Heart rate**


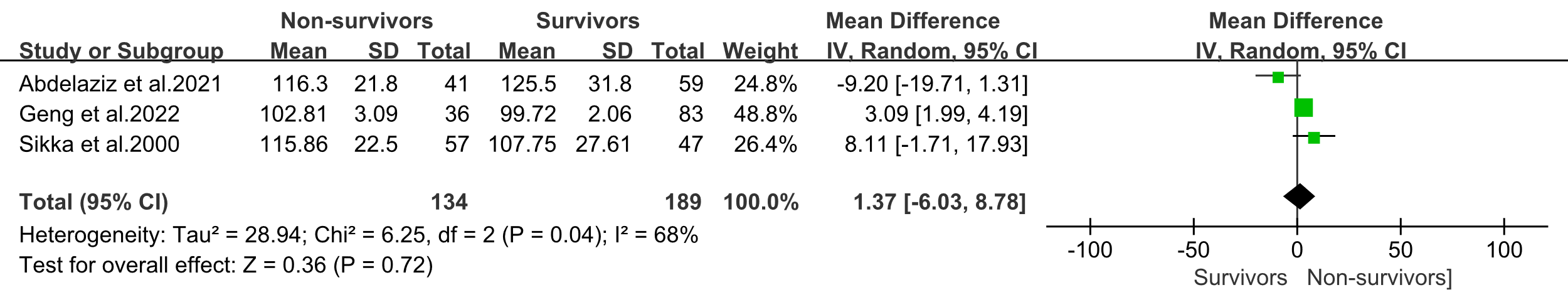


**(C) Body temperature**


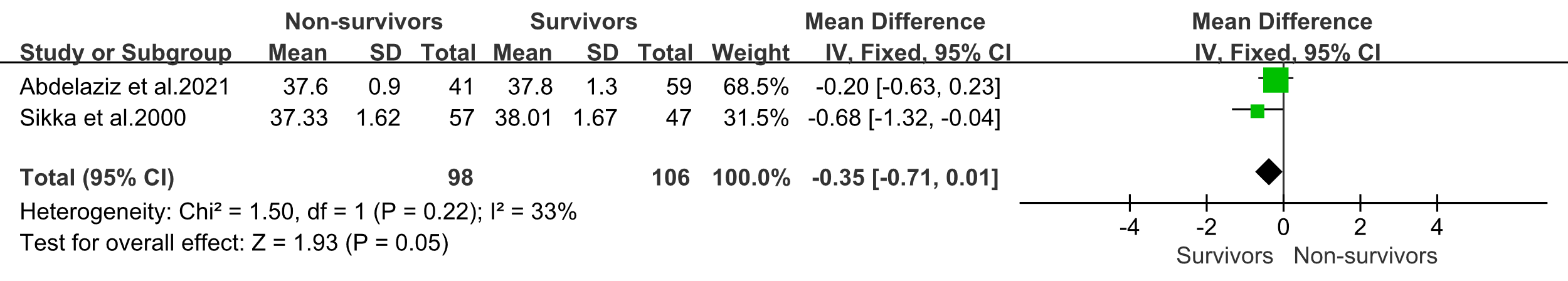


**(D) Mean arterial pressure (MAP)**


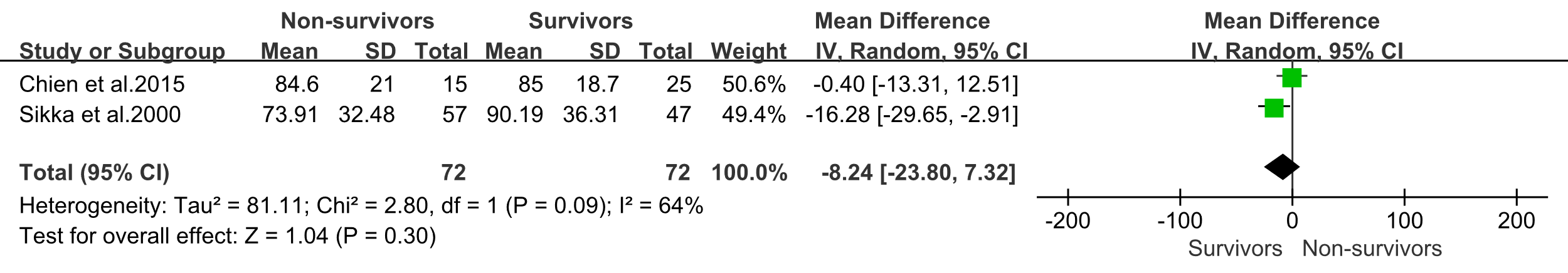


**(E) Urine output**

**
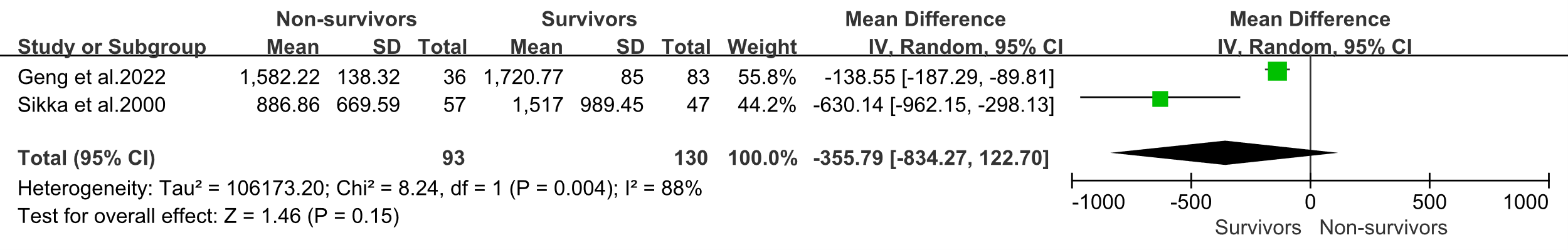
**

**Supplemental Figure 2:** Association between the comorbidities and severe pneumonia mortality.

**(A) Chronic obstructive pulmonary disease (COPD)**


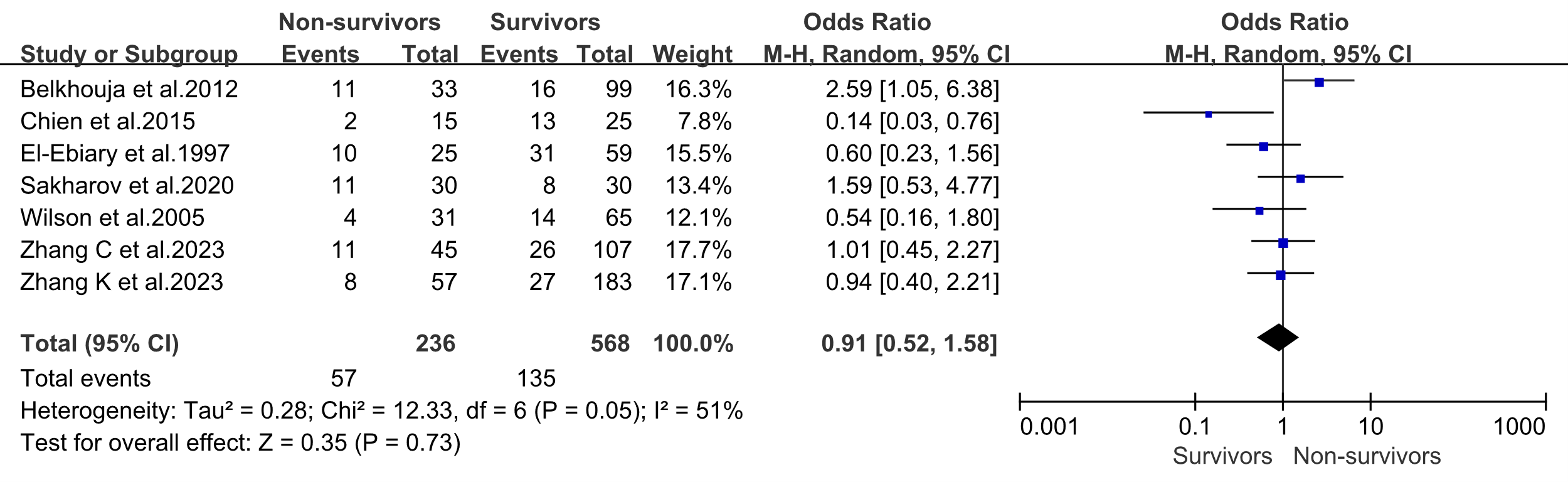


**(B) Hypertension**


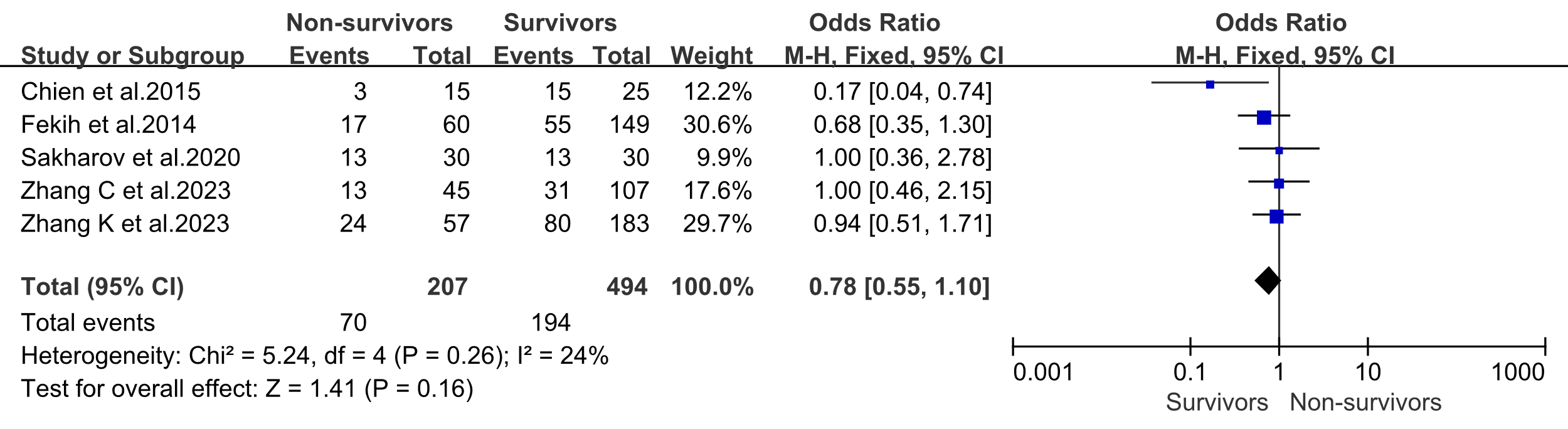


**(C) Diabetes mellitus**


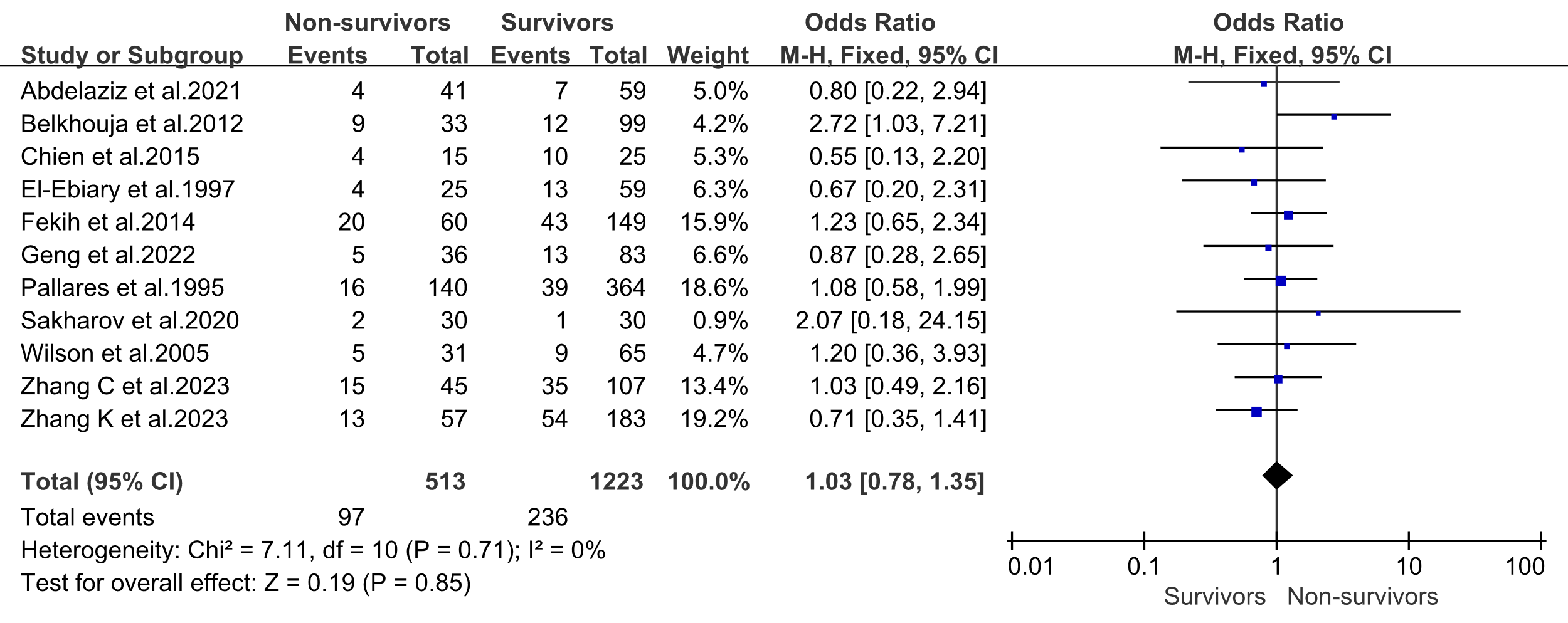


**Supplemental Figure 3:** Association between the complications and severe pneumonia mortality.

**(A) Respiratory failure**

**
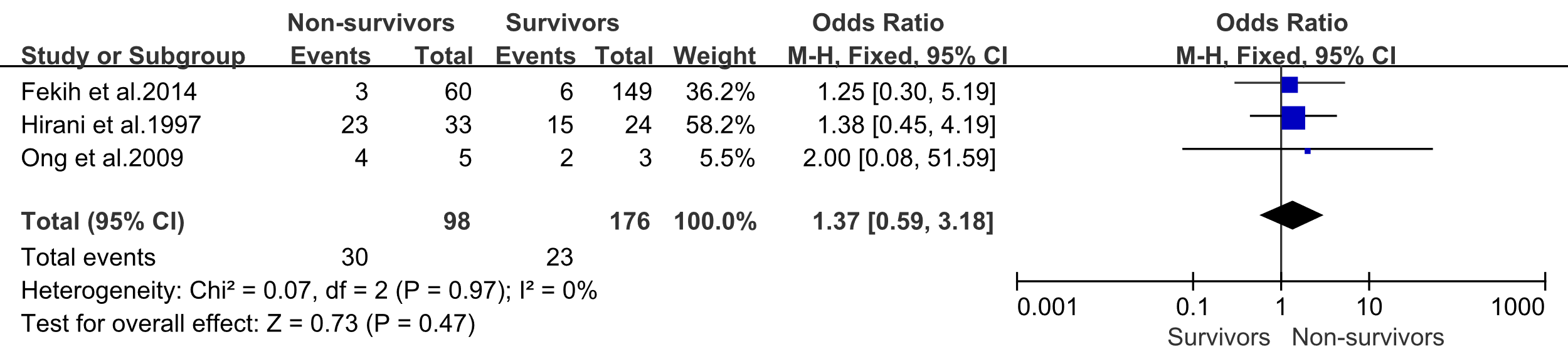
**

**(B) Acute confusion**


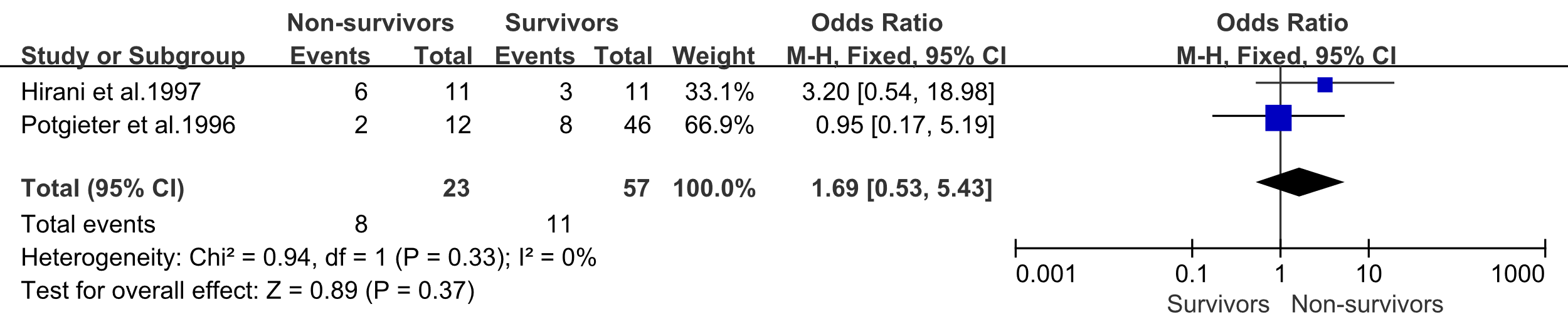


**(C) Pleural effusion**


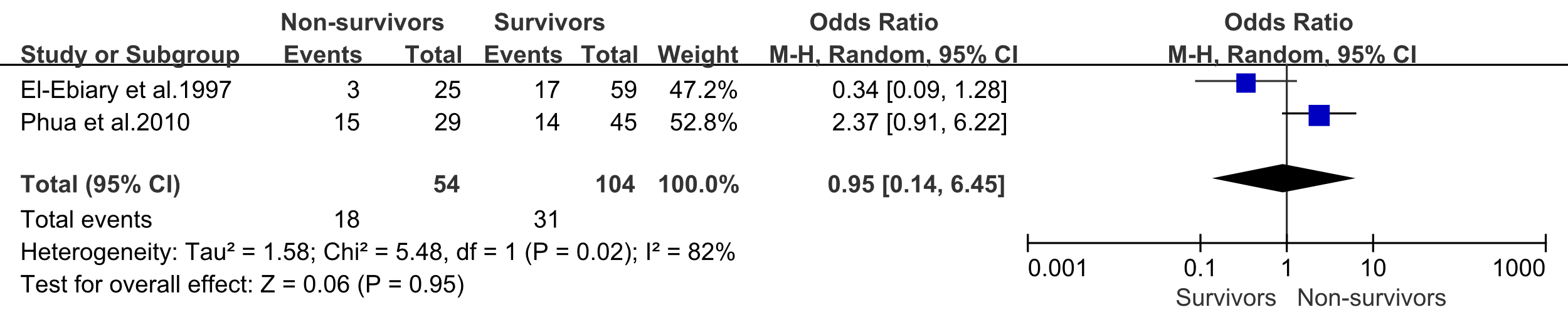


**(D) Bacteremia**

**
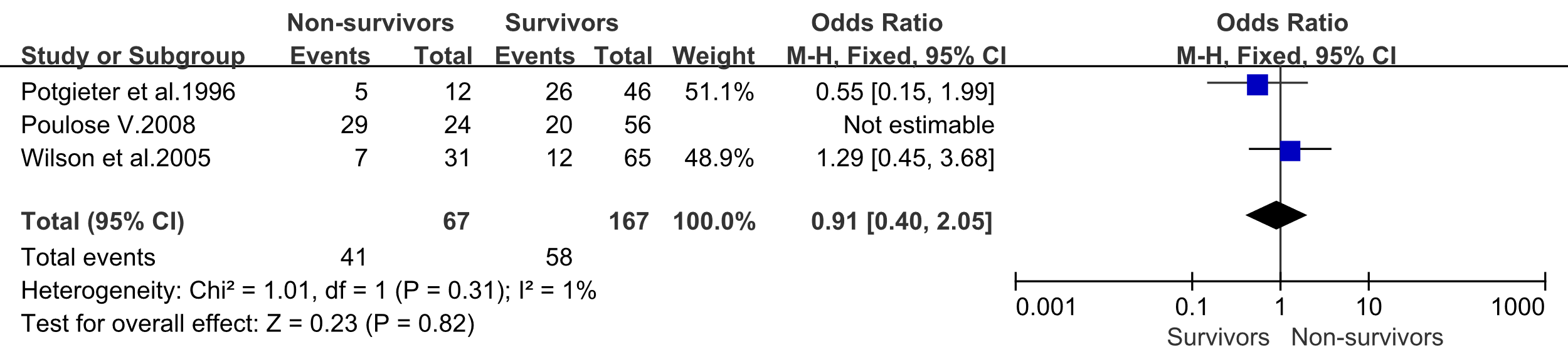
**

**Supplemental Figure 4:** Association between the laboratory results and severe pneumonia mortality.

**(A) Bacterial mixed infection**

**
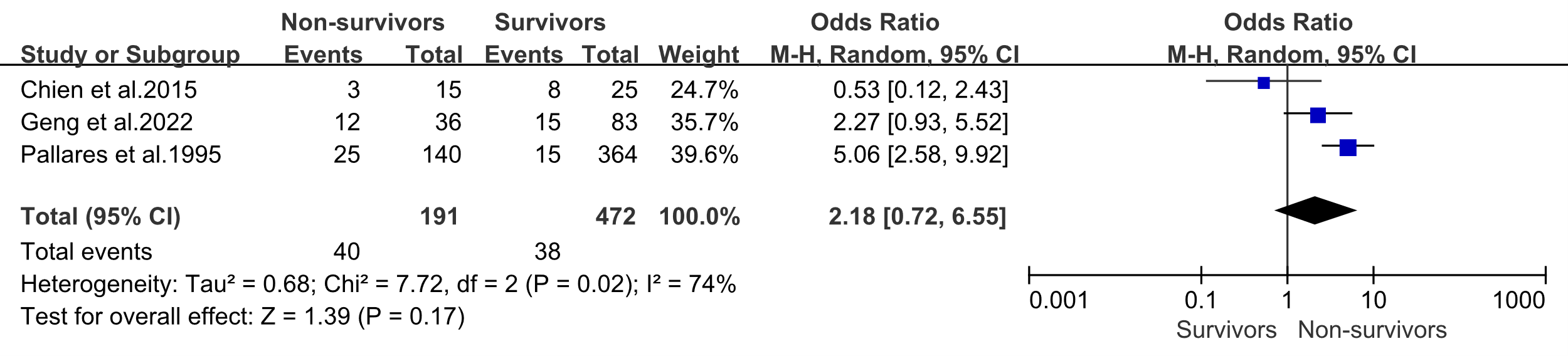
**

**(B) Positive blood culture**

**
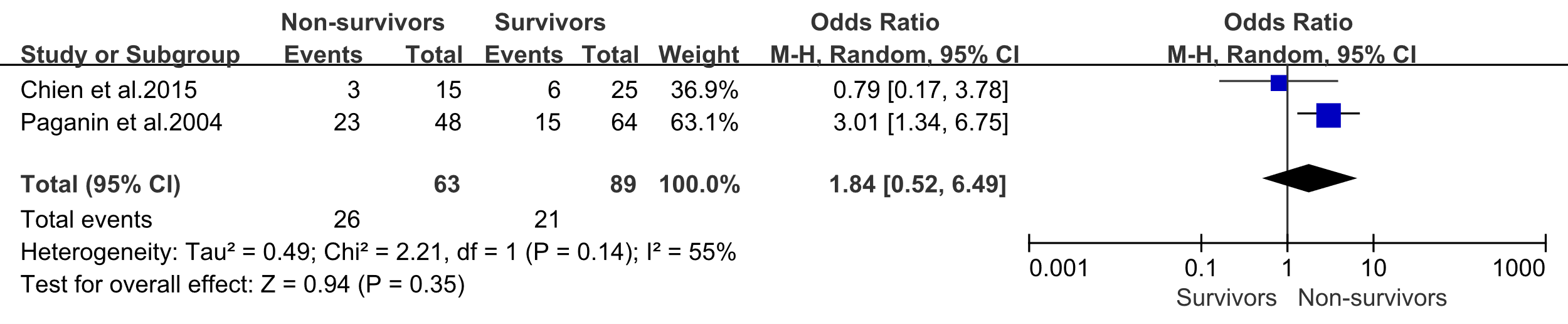
**

**(C) Sputum cont culture growth**

**
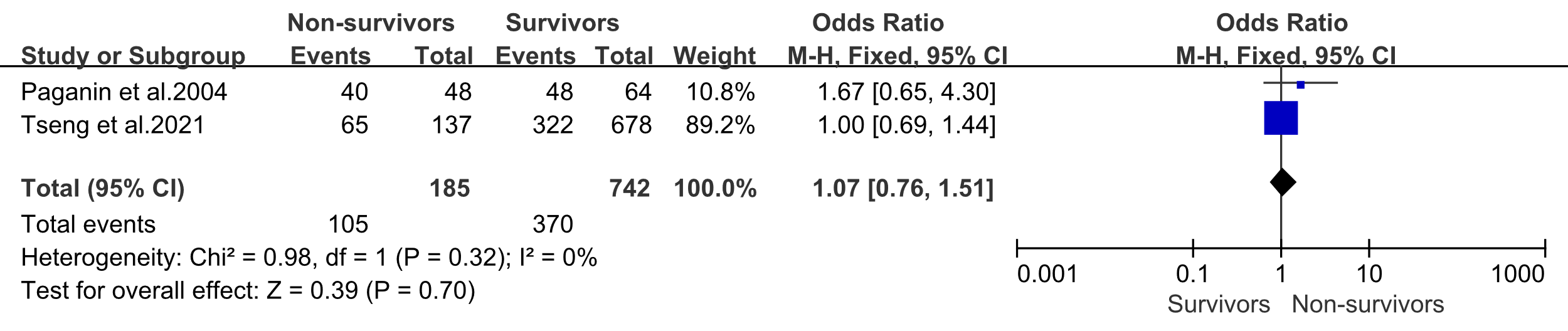
**

**(D) Gram-positive microorganism**

**
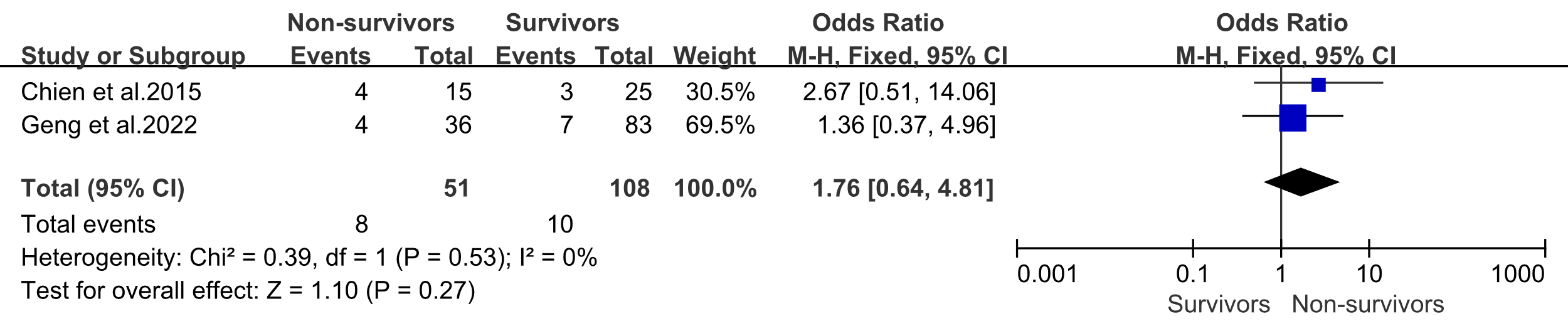
**

**(E) White blood cell (WBC)**

**
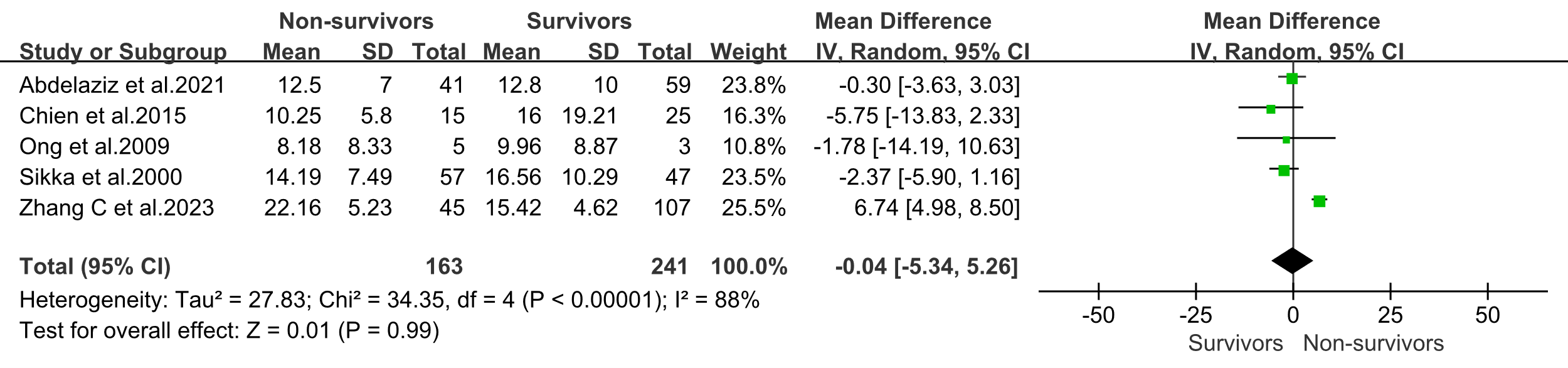
**

**(F) Platelet count**

**
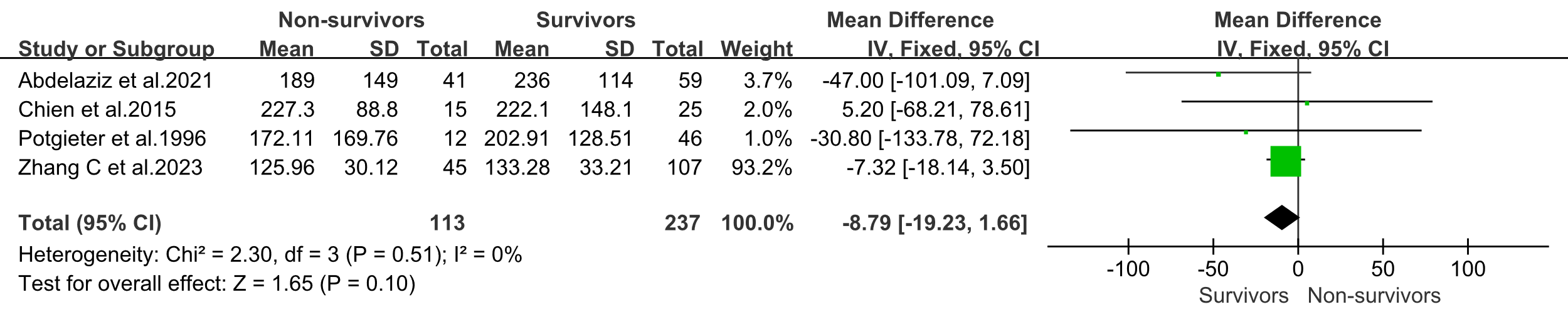
**

**(G) Hemoglobin**

**
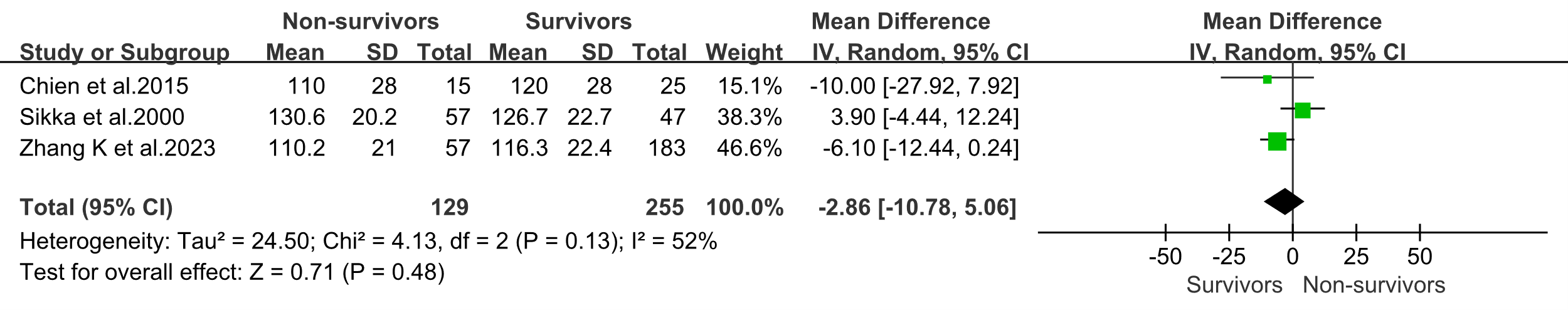
**

**(H) Glutamic oxaioacetic transaminase (AST)**


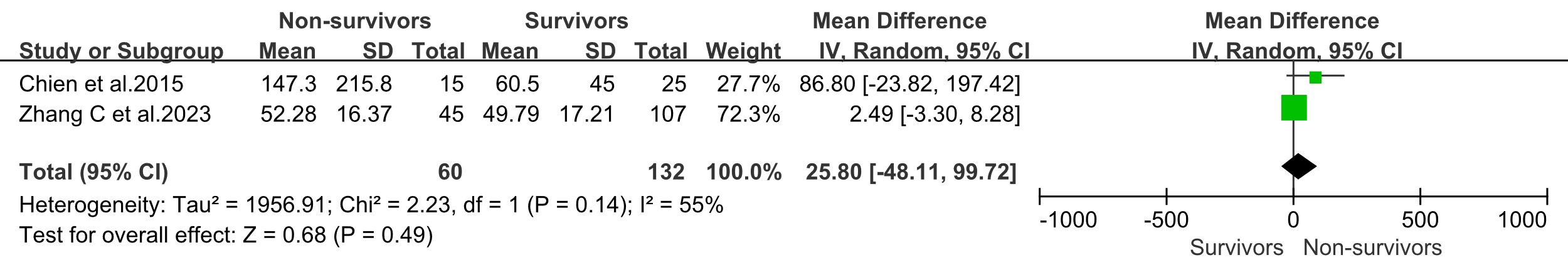


**(I) Potassium**

**
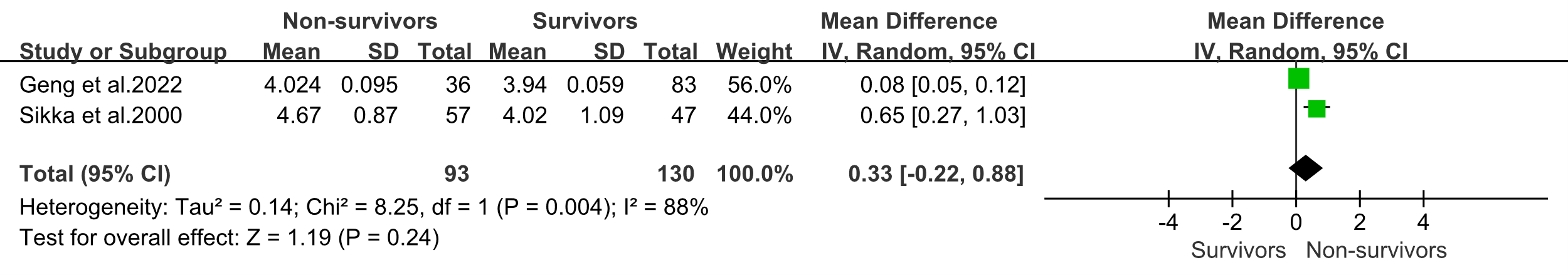
**

**Supplemental Figure 5:** Association between the long-term prognosis outcomes and severe pneumonia mortality.

**(A) Length of hospital stay**

**
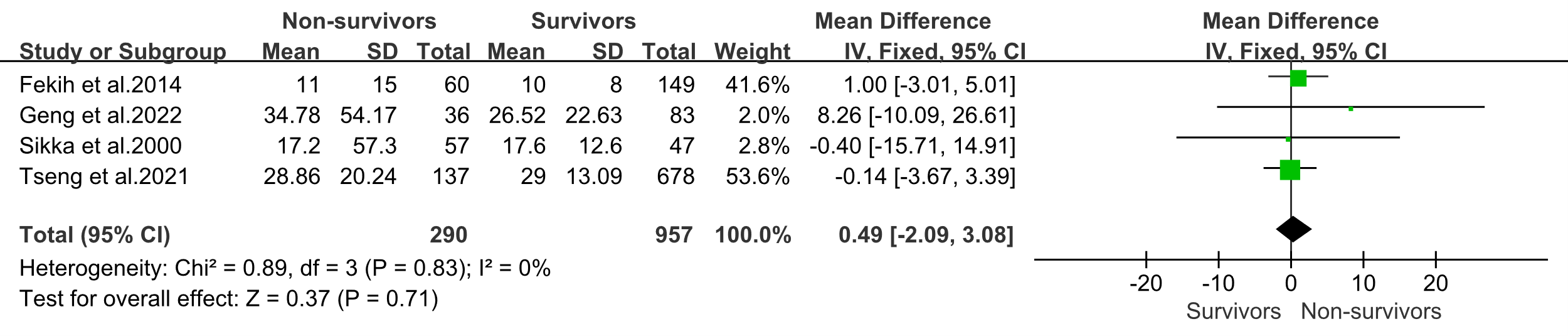
**

**(B) Length of ICU stay**

**
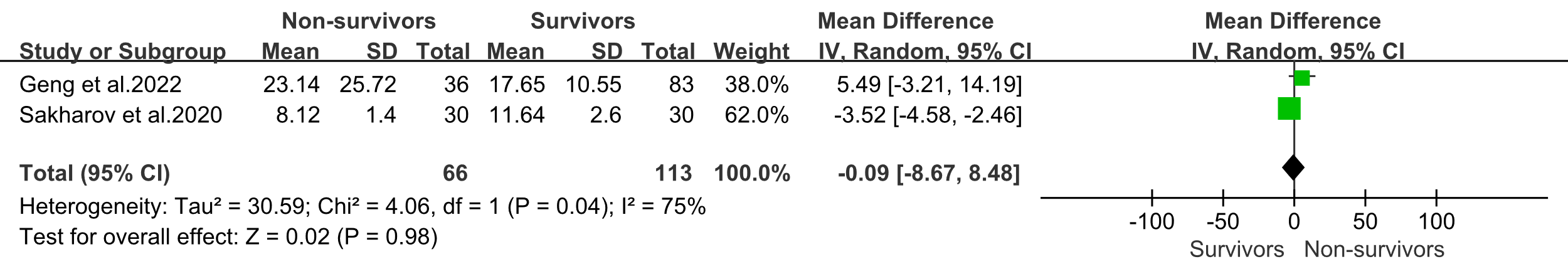
**
